# Prevalence and clinical characteristics of perinatal chronic lung disease by infant gestational age

**DOI:** 10.3233/NPM-200412

**Published:** 2021-02-04

**Authors:** K. Mavunda, X. Jiang, C.S. Ambrose

**Affiliations:** a Kidz Medical Services, Coral Gables, FL, USA; b EpidStat Institute, Ann Arbor, MI, USA; c AstraZeneca, Gaithersburg, MD, USA

**Keywords:** Bronchopulmonary dysplasia, respiratory syncytial virus, immunoprophylaxis

## Abstract

**BACKGROUND:**

Children with perinatal chronic lung disease (CLD) are at elevated risk for severe respiratory syncytial virus (RSV) disease in the first two years of life. The American Academy of Pediatrics policy does not recommend RSV immunoprophylaxis for infants with CLD born at ≥32 weeks’ gestational age (wGA). The objective of this study was to describe the number and clinical characteristics of US infants in this population.

**METHODS:**

Birth hospitalization data from the Kids’ Inpatient Database were utilized to estimate the prevalence of CLD (International Classification of Diseases, Ninth Revision [ICD-9] = 770.7) in 2003-2012 overall and by gestational age (ICD-9 = 765.21–765.29). CLD birth hospitalizations were evaluated by length of stay, costs, ventilatory support, and inpatient mortality.

**RESULTS:**

A total of 33,537 infants were diagnosed with CLD, representing 0.2% of US births; 79% had wGA coded in the database. Among infants with CLD with wGA, 3.5% were born at >32 wGA, representing 7 of every 100,000 US births, or approximately 300 infants annually. Across all wGA categories, birth hospitalization length of stay and costs were elevated, and mechanical ventilation use ranged from 73% to 97%. All-cause inpatient mortality was highest among those <27 wGA and >32 wGA.

**CONCLUSIONS:**

Approximately 300 infants born at >32 wGA are diagnosed with CLD annually in the United States. The all-cause perinatal mortality rate is high in this population. The rationale for excluding this small but high-risk group of infants from the recommendations for RSV immunoprophylaxis is unclear.

## 1 Background

Perinatal chronic lung disease (CLD), previously referred to as bronchopulmonary dysplasia (BPD), is associated with preterm birth, occurring rarely among term infants. Children with CLD are at elevated risk for severe respiratory syncytial virus (RSV) disease in the first two years of life [1]. Definitions of CLD/BPD identify infants who require supplemental oxygen at 28 days of life or 36 weeks’ postmenstrual age, with no restriction by gestational age at birth [2, 3]. However, the American Academy of Pediatrics (AAP) Committee on Infectious Diseases (COID) 2014 guidance does not recommend RSV immunoprophylaxis for infants with CLD born at ≥32 weeks’ gestational age (wGA), even though infants with CLD/BPD up to 41 wGA were included in pivotal efficacy studies [4]. No rationale for the exclusion of infants with CLD born at ≥32 wGA was provided by the AAP COID; however, it may relate to concerns regarding the overdiagnosis or severity of CLD in infants born at later gestational age. This analysis aimed to describe the prevalence and birth hospitalization characteristics of US infants diagnosed with CLD as a function of wGA at birth. Birth hospitalization characteristics were summarized to provide insight into the overall health status of these infants. It was not possible to examine the characteristics of post-discharge RSV hospitalizations among infants with CLD by wGA because wGA is not routinely coded for infant RSV hospitalizations.

## 2 Methods

The Kids’ Inpatient Database (KID) is a nationally representative, publicly available survey conducted every 3 years in the United States [5]. Birth hospitalization data (International Classification of Diseases, Ninth Revision [ICD-9] codes V30.XX–V39.XX) from KID were utilized to estimate the prevalence of CLD (ICD-9 = 770.7: chronic respiratory disease arising in the perinatal period) among US infants in 2003 to 2012 overall and as a function of coincident codes for gestational age (ICD-9 = 765.21–765.29, reported in 2-week intervals). KID data from 2015 were not available due to the transition to ICD-10.

The data were previously collected and statistically de-identified and are compliant with the de-identification conditions set forth in Sections 164.514 (a)-(b)1ii of the Health Insurance Portability and Accountability Act of 1996 Privacy Rule. The provisions in the Privacy Rule allow for use of health information that neither identifies nor provides a reasonable basis to identify an individual; therefore, approval from an institutional review board was not sought.

Because ICD-9 Clinical Modification (CM) codes combine 31 and 32 wGA infants, our analyses focused on infants born at >32 wGA. CLD birth hospitalizations were described for each year of KID availability (2003, 2006, 2009, 2012) and all years combined by length of stay, total cost (2015 US$), proportion requiring ventilatory support during the birth hospitalization, and proportion of inpatient mortality [6]. Ventilatory support (mechanical ventilation and/or continuous positive airway pressure [CPAP] ventilators) was based on ICD-9-CM procedure codes 93.90, 96.01, 96.02, 96.03, 96.04, 96.05, 96.70, 96.71, or 96.72 (Table 1).

**Table 1:** ICD-9-CM Procedure Codes for Ventilatory Support

ICD-9-CM Procedure Code	Description	Invasive vs Non-invasive
93.90	Non-invasive mechanical ventilation	Non-invasive
96.01	Insertion of nasopharyngeal airway	Non-invasive
96.02	Insertion of oropharyngeal airway	Non-invasive
96.03	Insertion of esophageal obturator airway	Non-invasive
96.04	Insertion of endotracheal tube	Invasive
96.05	Other intubation of respiratory tract	Invasive
96.70	Continuous invasive mechanical ventilation of unspecified duration	Invasive
96.71	Continuous invasive mechanical ventilation for less than 96 consecutive hours	Invasive
96.72	Continuous invasive mechanical ventilation for 96 consecutive hours or more	Invasive

ICD-9-CM, International Classification of Diseases, Ninth Edition, Clinical Modification.

Univariate logistic regression was used to examine temporal trends in mechanical ventilation and inpatient mortality. Because length of hospitalization and total hospitalization charges are not normally distributed, geometric means were calculated. Cost estimates were inflation-adjusted to 2015 US$. Univariate linear regression was used to test temporal trends in length of hospitalization and total hospitalization charges. All data management and analyses for this study were performed using SAS/STAT software, version 9.4 of the SAS System (SAS Institute Inc.), with statistical procedures that incorporate weights to account for the structure of the sample survey data.

## 3 Results

**Fig. 1:**
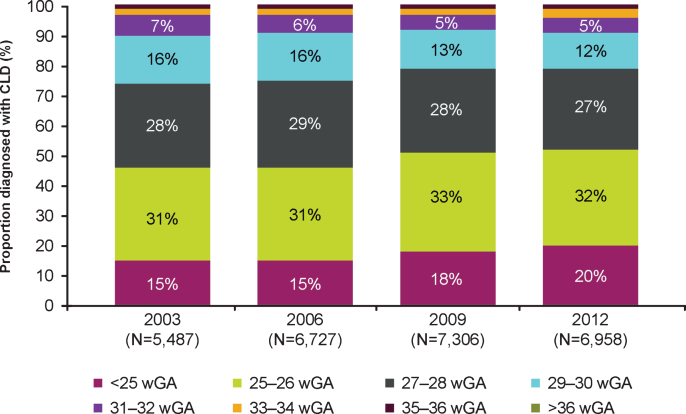
Distribution of US infants diagnosed with CLD by gestational age by year. **Abbreviations:** CLD, chronic lung disease; wGA, weeks’ gestational age. Proportion of >36 wGA infants was nearly 0.

A total of 33,537 infants had a CLD diagnosis across the 4 years for which data were available, representing 0.2% of US births. Characteristics of infants with birth hospitalization diagnosis of CLD are shown (Table 2). The prevalence of perinatal CLD declined from 21.9 to 20.2 per 10,000 between 2003 and 2012. A total of 26,477 infants with CLD (79%) had wGA coded in the database for their birth hospitalization (Table 2). Among those with wGA coded, the percentage of infants with CLD born at <27 wGA increased from 45% in 2003 to 52% in 2012 (Fig. 1). An estimated 3.5% of infants with CLD with wGA coded were born at >32 wGA, representing seven of every 100,000 US births, or approximately 300 infants annually. In a sensitivity analysis, 2.7% of all infants with CLD (regardless of whether wGA was coded) had a code for birth at >32 wGA, representing 6 of every 100,000 US births.

**Table 2:** Characteristics of Infants With Birth Hospitalization Diagnosis of CLD, 2003-2012

	Birth Hospitalization With CLD			Newborn Hospitalization
	≤32 wGA	>32 wGA	All
Total	25,558	919	33,537	15,778,872
Raw count	18,326	658	23,775	4,152,180
Sex
Male, *n* (%)	14,025 (54.9)	572 (62.2)	18,538 (55.3)	8,055,994 (51.1)
Raw count	10,076	408	13,158	2,208,076
Female, *n* (%)	11,530 (45.1)	348 (37.8)	14,994 (44.7)	7,692,471 (48.8)
Raw count	8248	250	10,614	1,938,758
Race
White, *n* (%)	9769 (38.2)	438 (47.6)	12,527 (37.4)	6,590,799 (41.8)
Raw count	6986	312	8869	1,725,313
Black, *n* (%)	6077 (23.8)	132 (14.4)	7729 (23.0)	1,709,784 (10.8)
Raw count	4292	91	5385	515,601
Hispanic, *n* (%)	3580 (14.0)	134 (14.5)	4589 (13.7)	2,866,180 (18.2)
Raw count	2604	98	3291	735,631
Asian or Pacific Islander, *n* (%)	685 (2.7)	21 (2.3)	826 (2.5)	597,405 (3.8)
Raw count	516	16	618	158,867
Native American, *n* (%)	148 (0.6)	NA	181 (0.5)	91,774 (0.6)
Raw count	109	NA	133	24,339
Other, *n* (%)	1353 (5.3)	36 (3.9)	1660 (4.9)	764,726 (4.8)
Raw count	981	27	1191	204,288
Admission quarter
January – March, *n* (%)	5688 (22.3)	174 (19.0)	7378 (22.0)	3,548,223 (22.5)
Raw count	4121	126	5285	936,176
April – June, *n* (%)	6066 (23.7)	216 (23.5)	8024 (23.9)	3,650,261 (23.1)
Raw count	4395	154	5746	964,915
July – September, *n* (%)	5886 (23.0)	212 (23.0)	7808 (23.3)	3,918,697 (24.8)
Raw count	4258	157	5576	1,030,160
October – December, *n* (%)	5788 (22.6)	233 (25.4)	7735 (23.1)	3,684,534 (23.4)
Raw count	4171	167	5503	970,795
Type of health insurance^*a*^
Private, *n* (%)	11,613 (45.4)	451 (49.1)	15,065 (44.9)	7,751,220 (49.1)
Raw count	8317	320	10,673	2,008,336
Other, *n* (%)	13,932 (54.5)	467 (50.7)	18,435 (55.0)	7,998,209 (50.7)
Raw count	10,000	337	13,076	2,136,507

CLD, chronic lung disease; wGA, weeks’ gestational age. Missing values are not reported. NA, *n* is less than or equal to 10. ^a^Private: Private including health maintenance organization.

**Fig. 2:**
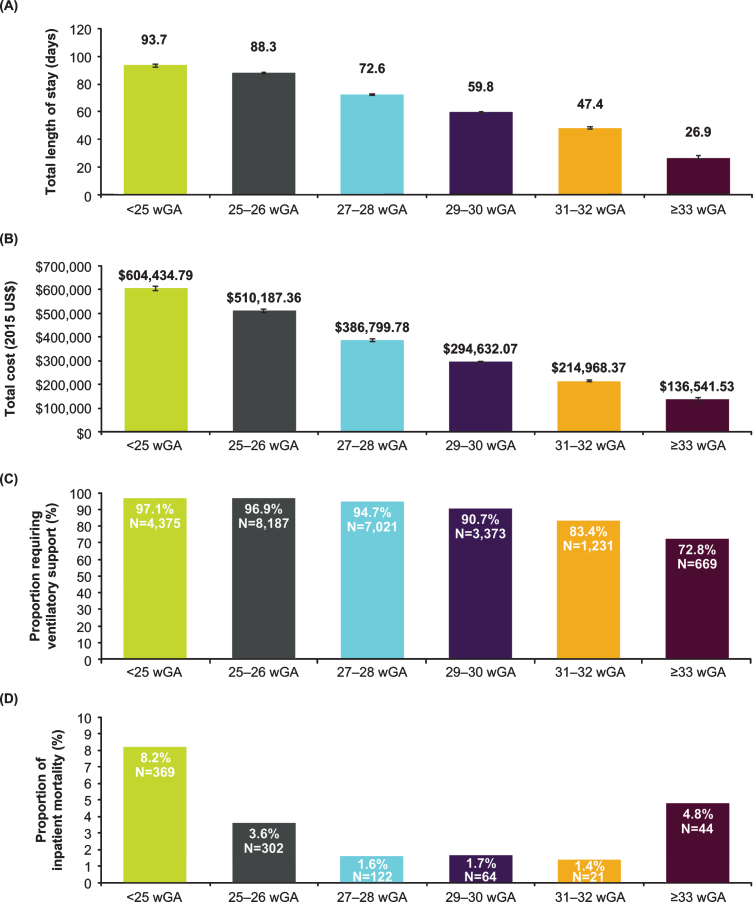
Characteristics of birth hospitalizations of infants with CLD by gestational age in the KID, 2003-2012. (A) Length of stay in days, geometric mean±SE; (B) Total charge adjusted to 2015 US$, geometric mean±SE; (C) Ventilatory support; (D) Inpatient mortality. **Abbreviations:** CLD, chronic lung disease; KID, Kids’ Inpatient Database; wGA, weeks’ gestational age.

Among infants with CLD, birth hospitalization length of stay and hospital charges were high across all wGA groups and trended higher among those born at earlier wGA (Fig. 2A and 2B). Across all wGA categories of infants with CLD, mechanical ventilation use was elevated at 73% to 97% of birth hospitalizations, and use was greatest among those born at earlier wGA (Fig. 2C). Most infants with CLD with mechanical ventilation required invasive ventilation, with the proportion treated with non-invasive ventilation ranging from ≤2% of infants born at <27 wGA to 11.3% of infants born at 31 to 32 wGA. All-cause inpatient mortality ranged from 1.4% to 8.2% of infants with CLD and followed a U-shaped distribution, with a higher frequency among those <27 wGA and >32 wGA (Fig. 2D).

**Fig. 3:**
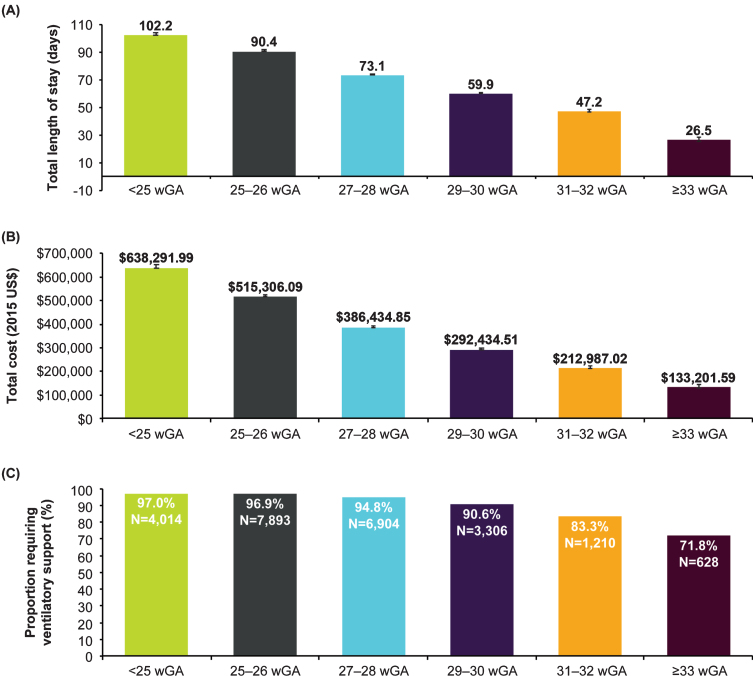
Characteristics of birth hospitalizations of infants with CLD excluding infants who died prior to hospitalization discharge by gestational age in the KID, 2003-2012. (A) Length of stay in days, geometric mean±SE; (B) Total charge adjusted to 2015 US$, geometric mean±SE; (C) Ventilatory support. **Abbreviations:** CLD, chronic lung disease; KID, Kids’ Inpatient Database; wGA, weeks’ gestational age.

When analyzed by year, birth hospitalization length of stay and hospital charges (in 2015 US$) increased over time; differences were significant (*p* < 0.001) for all wGA cohorts except for infants with CLD who were born at >32 wGA (Table 3). Mechanical ventilation use remained consistent over time, and changes were not significant. Inpatient mortality declined over time among infants with CLD born at <25 wGA (*p* < 0.016) but was similar across years for all other wGA categories. Data regarding birth hospitalizations of infants with CLD that excluded infants who died prior to hospitalization discharge were similar (Fig. 3).

**Table 3:** Characteristics of Birth Hospitalizations of Infants With CLD by Gestational Age in the KID by Individual Years, 2003–2012

	Ventilatory Support, %					Length of Stay,^*a*^ days					Total Charge,^*a*,*b*^ 2015 US$					Inpatient Mortality, %				
	2003	2006	2009	2012	*P* Value	2003	2006	2009	2012	*P* Value	2003	2006	2009	2012	*P* Value	2003	2006	2009	2012	*P* Value
CLD <25 wGA	97.2	97.4	95.8	98.1	0.555	80.5	89.8	96.6	102.3	<0.001	434,599.7	492,606.7	617,421.2	796,764	<0.001	11.0	9.0	7.6	6.6	0.016
CLD 25–26 wGA	96.4	97.0	96.9	97.1	0.450	85.1	84.3	89.2	94.0	<0.001	410,381.3	449,236.9	526,786.2	644,491.8	<0.001	3.1	3.8	3.8	3.4	0.724
CLD 27–28 wGA	95.0	94.6	95.3	94.1	0.560	66.7	70.0	75.0	78.1	<0.001	302,553.2	349,703.9	416,895.8	479,533.1	<0.001	1.7	1.3	1.8	1.7	0.759
CLD 29–30 wGA	90.9	90.9	91.0	90.1	0.707	55.4	57.6	62.4	64.5	<0.001	229,823.5	279,486.8	330,176.2	353,753.4	<0.001	2.1	1.5	2.0	1.2	0.464
CLD 31–32 wGA	86.1	82.1	86.8	78.9	0.125	45.2	45.3	46.9	53.3	<0.001	169,346.2	209,786.1	231,650.9	265,906.3	<0.001	NA	NA	NA	NA	NA
CLD >32wGA	76.5	75.4	68.9	70.0	0.098	26.1	29.2	24.2	28.5	0.898	112,295.3	137,916.8	140,256.4	160,492.5	0.044	5.0	5.6	NA	NA	NA

CLD, chronic lung disease; KID, Kids’ Inpatient Database; wGA, weeks’ gestational age. ^a^Geometric mean. ^b^Adjusted to 2015 US$. NA, *n* is less than or equal to 10.

## 4 Discussion

Using the large, population-based sample provided by KID, this study is the first to demonstrate that approximately 300 infants annually are born at >32 wGA and diagnosed with CLD in the United States. Although these infants born at >32 wGA on average have shorter birth hospitalizations that cost less than those of infants with CLD born at <32 wGA, their birth hospitalizations are consistent with a high-risk status relative to otherwise healthy newborns [7]. The observation that infants with CLD born at later wGA have an elevated risk of all-cause death warrants further study; it may be due to a higher prevalence of comorbid conditions such as birth defects, respiratory infections, failure to thrive, or gastrointestinal disease.

Although not all preterm infants experience complications, preterm birth can result in short-term and long-term health consequences, including the incidence of CLD. An infant born at 29 to <32 wGA is considered very preterm, and according to the 2014 AAP guidance, qualifies for RSV immunoprophylaxis if the patient has a diagnosis of CLD [8]. Despite being more developed, moderate- and late-preterm infants born at 32 to 34 and 35 to 36 wGA can still experience incomplete respiratory system maturation, as development is generally not complete until the final weeks of pregnancy [9]. Additionally, infants born preterm have lower levels of maternal antibody that can provide protection against respiratory pathogens [10]. As a result, these moderate- and late-preterm infants also have increased risks of complications and infections that may require ventilatory or oxygen support, which in turn can lead to the development of CLD. Although rare, the incidence of neonatal complications and infections among term infants can similarly result in CLD [11].

Moderate- to late-preterm infants are physiologically and metabolically immature and have high rates of mortality and morbidity during the birth hospitalization [12–16]. During the initial birth hospitalization, late-preterm infants are four times more likely than term infants to have at least one medical condition diagnosed and are 3.5 times more likely to have at least two conditions diagnosed [12, 17]. In addition, these infants are more likely to be evaluated for sepsis, receive antibiotics, require ventilator support, and have prolonged hospitalization, which can negatively affect the pulmonary system [12, 15]. Existing data have also demonstrated that these infants are at higher risk of apnea, hypothermia and temperature instability, hypoglycemia, and hyperbilirubinemia than term infants [17–20]. Despite this, late-preterm infants have not been studied as intensely as more premature infants, and our understanding of their developmental biology remains incomplete [12].

Immature cardiovascular function due to delayed ductus arteriosus closure may complicate recovery of the preterm infant with respiratory distress and persistent pulmonary hypertension. Cardiac problems can also secondarily affect the pulmonary system [21]. Moreover, late-preterm infants may have immature gastrointestinal function in which feeding difficulties are associated with relatively low oromotor tone and problems with suck-swallow coordination [22]. When feeds are increased, immature gastrointestinal function may lead to problems with necrotizing enterocolitis [15, 23]. This can lead to cessation of oral feeds and the use of antibiotics for longer periods and can secondarily affect the pulmonary system [15]. Feeding problems may lead to aspiration into the lungs and subsequent pulmonary complications [24]. The situations described above that result in pulmonary compromise may lead to an increased need for oxygen support and/or ventilatory support; preterm infants who have these requirements often develop CLD [25].

Late-preterm infants may also have congenital abnormalities of the airway and lung parenchyma such as tracheoesophageal fistula, congenital diaphragmatic hernia, and pulmonary hypoplasia [26]. Although these abnormalities may lead to CLD, they are less common than the mechanisms described above. Advances in medical knowledge and surgical skills have led to decreases in morbidity from these congenital abnormalities, and thus the development of CLD in late-preterm infants is less likely; therefore, they would not have been included in this analysis [27]. Clinicians have been using palivizumab, the only available RSV immunoprophylaxis, in patients with congenital airway anomalies and pulmonary abnormalities for many years [7, 28].

Infants with CLD at any gestational age are more likely to have complications requiring hospitalization when infected with RSV [1]. The current study does not provide data on the risk of RSV among infants with CLD as a function of gestational age. However, we believe that the evidence suggests that all infants with CLD, regardless of gestational age at birth, warrant special medical attention and are likely to benefit from increased efforts to reduce the incidence of severe respiratory infections from pathogens like RSV.

Regarding the limitations of this study, it should be noted that the administrative claims data collected by KID are generated for billing purposes, not research. As a result, misclassification may have occurred due to coding errors or under- or over-coding. Furthermore, 21% of infants with CLD did not have a code for wGA; to address this gap, we applied the distribution of wGA codes among those with a wGA code to the entire cohort, with a sensitivity analysis reporting prevalence estimates without any imputation; both analyses yielded similar prevalence estimates. Regarding birth hospitalization characteristics, if infants without a wGA code were systematically different than those with a wGA code, current results could represent skewed estimates. However, given that 79% did have a wGA code, it is unlikely that any difference would be significant enough to meaningfully alter our estimates. The study definition of ventilatory support was purposefully broad, and for this reason, we examined invasive and non-invasive ventilation separately. Additionally, the more procedurally oriented codes (9601, 9602, 9603, 9604, 9605) were infrequently found in isolation; of the 24,856 infants with non-missing gestational age diagnosed with CLD and with ventilatory support, only 618 were identified as requiring ventilatory support based on these codes alone. We did not examine which comorbid diagnoses were more prevalent in ≥32 wGA infants diagnosed with CLD, including those who died; that question would benefit from further examination in future analyses of neonatal-specific datasets.

In summary, approximately 300 infants are born at >32 wGA and diagnosed with CLD annually in the United States. The all-cause perinatal mortality rate in infants with CLD born at >32 wGA is high, which is consistent with these infants being at higher risk for comorbidities. Although only a small number of infants are born at >32 wGA and diagnosed with CLD, we believe that there is no clear rationale for excluding this Food and Drug Administration (FDA)-approved high-risk group from the populations recommended for RSV immunoprophylaxis.

## Disclosure statements

This research was funded by AstraZeneca, the manufacturer of palivizumab. The sponsor was involved in the design and conduct of the study and approval of the manuscript. Decision rights about content belonged collectively to the authors and not to the sponsor. No substantive changes were made to the manuscript as a result of the company review. KM is on the speakers bureau for AstraZeneca. XJ provided consultant/research support for AstraZeneca. CSA is an employee of AstraZeneca.
